# A Pharmacokinetic and Bioavailability Study of *Ecklonia cava* Phlorotannins Following Intravenous and Oral Administration in Sprague–Dawley Rats

**DOI:** 10.3390/md22110500

**Published:** 2024-11-04

**Authors:** Hyeon-Cheol Shin, Clint Rosenfeld, Robert J. Guttendorf, Susan B. Wade, Yong Ju Park, Ju Hee Kim, Seong Ho Kim, Bong Ho Lee, Hye Jeong Hwang

**Affiliations:** 1 Phloronol Inc., 490 Post Street, Suite 1700, San Francisco, CA 94102, USA; sbbwade@gmail.com; 2 Center for Molecular Intelligence, The State University of New York Korea, Incheon 21985, Republic of Korea; 3 MPI Research Inc., 54943 North Main Street, Mattawan, MI 49071, USA; clint.rosenfeld@crl.com; 4 Charles River, 54943 North Main Street, Mattawan, MI 49071, USA; 5 Aclario Inc., 45600 Terminal Drive, Suite 200, Sterling, VA 20166, USA; rguttendorf@aclairo.com; 6 Botamedi Inc., Cheomdan-ro 8-gil, Jeju 63309, Republic of Korea; bmrlpark@kgs.ne.kr (Y.J.P.); wngml0313@hepatall.com (J.H.K.); ksh4030001@hanmail.net (S.H.K.); 7 DA-JUNG Research & Development Center, 371, Jangsu-ro, Jincheon-gun 27819, Republic of Korea; 8 Hepatall Inc., 96, Cheomdan-ro 8-gil, Jeju 63309, Republic of Korea; 9 Department of Chemical and Biological Engineering, Hanbat National University, Daejeon 34158, Republic of Korea; lbh011@hanbat.ac.kr

**Keywords:** *Ecklonia cava*, phlorotannins, pharmacokinetics, bioavailability, dieckol, 8,8′-bieckol, phlorofucofuroeckol-A (PFF-A)

## Abstract

This study examines the pharmacokinetics and bioavailability of phlorotannins from *Ecklonia cava* in rats following intravenous and oral administration. Known for their potent antioxidant, anti-inflammatory and many other bioactivities, these phlorotannins, particularly dieckol, 8,8′-bieckol, and phlorofucofuroeckol-A (PFF-A), were analyzed using high-performance liquid chromatography coupled with tandem mass spectrometry. Intravenous administration at 10 mg/kg allowed detectability in plasma for up to 36 h for dieckol and 8,8′-bieckol, but only 2 h for PFF-A. Oral administration at doses of 100 mg/kg and 1000 mg/kg showed limited detectability, indicating low bioavailability and rapid clearance, particularly for PFF-A. The pharmacokinetic data suggest non-linear increases in the maximum plasma concentration (C_max_) and area under the curve (AUC) with increasing doses, pointing to significant challenges in achieving systemic availability of these eckols through oral administration. This study underscores the necessity for advanced formulation strategies and alternative routes of administration to enhance systemic bioavailability. At the same time, this result also suggests their effects may be through non-systemic pathways such as gut microbiome modulation or lipid-rich tissue targeting. The findings lay a crucial foundation for the further development of *Ecklonia cava* phlorotannins as therapeutic agents, offering insights into their pharmacokinetic behavior and informing enhancements in future clinical utility.

## 1. Introduction

**Figure 1:**
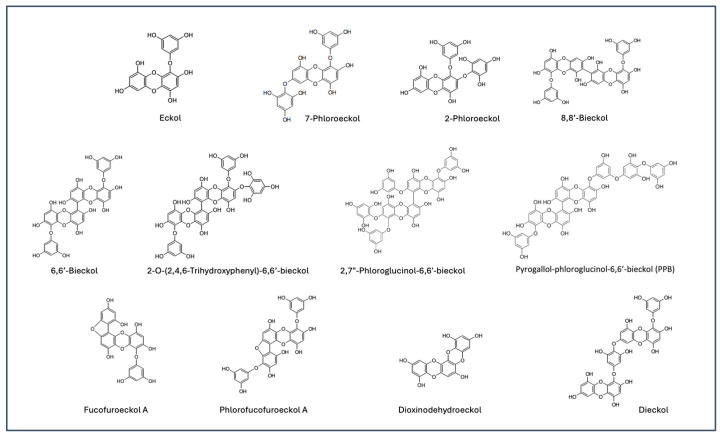
Various “eckol” subclass of phlorotannins naturally found in a brown alga *Ecklonia cava*.

Phlorotannins are phenolic metabolites primarily found in brown algae (Phaeophyta) and are composed of phloroglucinol units linked in various configurations [1,2,3]. Over the past two decades, phlorotannins have gained recognition as promising bioactive compounds with potential therapeutic properties, including antioxidant, anti-inflammatory, neuroprotective, UV-protective, antidiabetic, and anticancer activities [4,5,6,7,8,9], along with excellent safety profiles [10,11,12]. Among these, an oligomeric subclass called “eckols”—including eckol [13,14,15], 7-phloroeckol [16,17,18], dieckol [19,20], 6,6′-bieckol [21,22], 2,7-phloroglucinol-6,6′-bieckol [23,24], pyrogallol-phloroglucinol-6,6′-bieckol [25,26], 2-O-(2,4,6-Trihydroxyphenyl)-6,6′-bieckol [27,28], 8,8′-bieckol [29,30], dioxinodehydroeckol [31,32,33], fucofuroeckol A [34,35] and PFF-A [36,37,38,39,40]—are particularly noteworthy due to their unique structural feature of possessing one or two polyhydroxylated dibenzo-*p*-dioxin units, which are thought to contribute to their intriguing bioactivities and significant therapeutic potential (Figure 1).

Eckols, predominantly found in species such as *Ecklonia cava* (*E. cava*), *Ecklonia kurome*, *Ecklonia maxima*, *Ecklonia stolonifera*, *Eisenia arobrea*, and *Eisenia bicyclis*, have typically been extracted from these algae using organic solvents like methanol, ethanol, and ethyl acetate [41,42,43]. Recently, *E. cava* have gained substantial interest due to their high eckol content and potent bioactivities, making them applicable in a wide range of therapeutic uses [44]. For instance, both complexes and isolated forms of eckols derived from *E. cava* have demonstrated potent antioxidant [45,46], anti-inflammatory [13,21,47], antiviral [18,30,48,49], antidiabetic [20,50,51] and anticancer [14,22,52,53,54] properties. Furthermore, numerous studies have highlighted that individual eckols, as well as botanical extracts rich in these compounds, possess significant potential as disease-modifying therapeutic agents against various chronic degenerative diseases, including cardiovascular diseases [23,26,28,55], osteoarthritis [56,57,58], neurodegenerative diseases [59,60,61,62,63,64], and osteoporosis [65,66,67].

The safety profile of a standardized eckols-rich phlorotannin extract from *E. cava* has been comprehensively established through a series of GLP (good laboratory practice) studies, which include acute and chronic toxicity, genotoxicity, and other relevant safety assessments [10]. Additionally, the historical consumption data of *E. cava* have supported its regulatory authorizations as a new dietary ingredient in the United States [11] and as a novel food ingredient in the European Union [68].

Given this, an *E. cava* extract with a high content of eckols represents a highly safe botanical agent with significant potential for development into therapeutics for a wide range of diseases, particularly those where conventional drugs often fall short due to serious side effects.

Despite extensive research on the biological activities of eckols, little is known about their absorption, distribution, metabolism, and excretion (ADME) properties. This gap limits the ability to develop optimal dosing strategies, assess bioavailability, and predict human pharmacokinetics—critical elements in advancing these compounds from preclinical research to clinical applications.

Therefore, the primary objective of this study is to investigate, for the first time, the ADME properties of eckols in vivo. By generating key pharmacokinetic data, this study aims to provide the insights necessary for optimizing dosing regimens, assessing bioavailability, and predicting human pharmacokinetics, which are essential for translating eckol-based therapies from preclinical research to clinical settings for the treatment of chronic degenerative diseases.

To achieve this, we conducted a single-dose pharmacokinetic and bioavailability study in rats, evaluating systemic exposure to dieckol, 8,8′-bieckol, and PFF-A following intravenous and oral administration of an eckols-rich *E. cava* phlorotannin extract (EK-ECP). The three specific eckols were selected based on their abundance in the extract and their documented bioactivities, making them suitable candidates for pharmacokinetic evaluation. The findings from this study are expected to contribute valuable insights for the future development of eckol-based therapeutics.

## 2. Results

### 2.1. Chemical Analysis of EK-ECP

The total polyphenolic content of EK-ECP was measured by the Folin–Ciocalteu method as 91.0%. The phlorotannin nature of the polyphenol in EK-ECP was confirmed by ^1^H-NMR spectroscopic analysis. Furthermore, the individual phlorotannins in EK-ECP were identified by reverse-phase HPLC, with their corresponding isolated standards.

#### 2.1.1. 1H-NMR Analysis of EK-ECP

**Figure 2:**
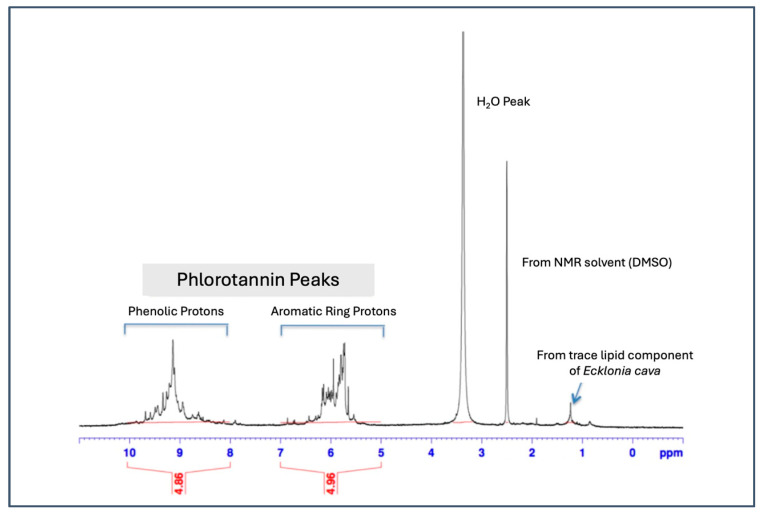
^1^H NMR spectrum of EK-ECP.

The ^1^H-NMR spectrum, provided in Figure 2, is consistent with the extract consisting primarily of phloroglucinol-based oligomers. Except for very minor peaks around 1 ppm, likely due to trace lipid components of *E. cava*, the predominant peaks observed at 5.5–6.5 ppm and 8.5–9.7 ppm correspond to the aromatic ring and phenolic hydroxyl protons from phloroglucinol units (Figure 2). The sharp peaks around 2.5 and 3.3 ppm are due to the protons from moisture and the solvent (DMSO) used for the NMR analysis. The broad nature of the ¹H-NMR peaks centered at 5.8 ppm and 9.2 ppm suggests a mixture of structurally similar phloroglucinol-based polymers, resulting from regioisomerism and varying degrees of polymerization.

#### 2.1.2. Identification of Individual Phlorotannins in EK-ECP

**Figure 3:**
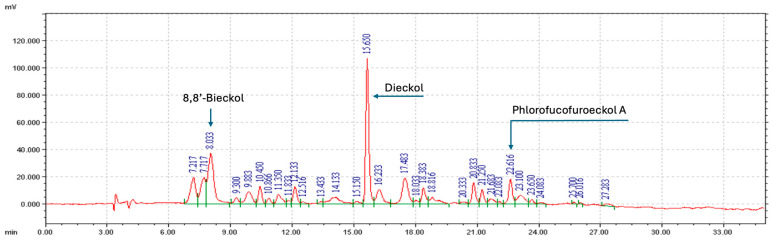
A typical HPLC chromatogram of EK-ECP with 91.0% phlorotannin content. The peaks of the three notable phlorotannins 8,8′-bieckol, dieckol, and PFF-A are notified.

A further detailed analysis of the phlorotannins present in EK-ECP was performed to identify and quantify the major components (individual phlorotannins) by reversed phase high performance liquid chromatography (RP-HPLC). A representative HPLC (Column: CAPCELL PAK C_18_; Detector: UV 234 nm) chromatogram and the identification of major components are presented in Figure 3. As shown in the chromatogram, dieckol at retention time 15.65 min was the most notable compound in EK-ECP. The chromatogram could be divided to relatively high (6–13 min), mid (13~20 min) and low polarity region (20~27 min) and each region could be represented by the regionally most notable peak 8,8′-bieckol, dieckol and PFF-A, respectively. These three phlorotannins were selected for monitoring the blood samples upon administration of EK-ECP during pharmacokinetic (PK) experiment. Other minor phlorotannins than the three notable ones identified were 2-O-(2,4,6-Trihydroxyphenyl)-6,6′-bieckol (7.217 min), 6,6′-bieckol (7.717 min), 7-phloroeckol (7.717 min), 2-phloroeckol (9.883 min), eckol (10.450 min), dioxinodehydroeckol (23.100 min) and fucofuroeckol A (23.650 min). Other notable peaks, such as those at 12.133, 17.483, 20.833, and 21.250 min, remain unidentified due to the technical challenges in isolating and identifying the corresponding single compounds. The corresponding structures of the identified phlorotannins are shown in Figure 1.

### 2.2. Pharmacokinetic Analysis

**Figure 4:**
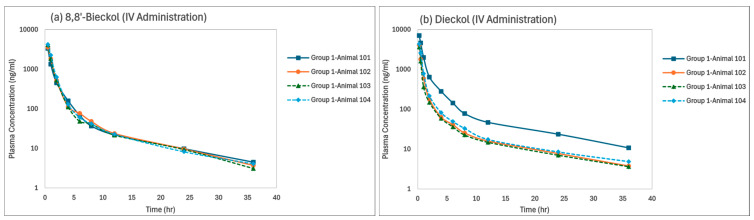

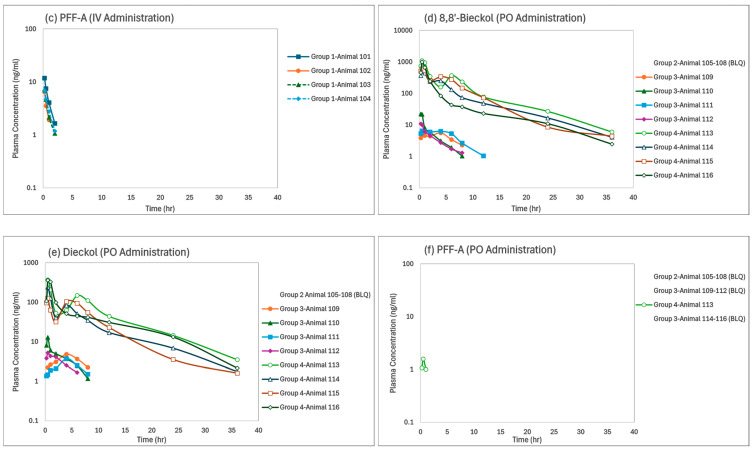
Plasma concentration–time profiles following IV and PO administration of 10, 100 or 1000 mg/kg EK-ECP to male rats: (**a**) 8,8′-bieckol (IV administration); (**b**) dieckol (IV administration); (**c**) PFF-A (IV administration); (**d**) 8,8′-bieckol (PO administration); (**e**) dieckol (PO administration); and (**f**) PFF-A (PO administration). BLQ represents “Below the limit of quantitation”.

EK-ECP was administered to male Sprague–Dawley rats by either IV bolus (Group 1, 10 mg/kg) or oral gavage (Groups 2, 3 or 4, at 10, 100 or 1000 mg/kg, respectively) administration. Blood collection intervals were pre dose (0), and 0.25, 0.5, 1, 2, 4, 6, 8, 12, 24 and 36 h post dose. Plasma samples were analyzed for concentrations of 8,8′-bieckol, dieckol, and PFF-A; all pre-dose sample results were less than the lower limit of quantitation (LLOQ < 1 ng/mL). The time profiles of the plasma concentrations of 8,8′-bieckol, dieckol, and PFF-A in individual animals are illustrated in Figure 4. The mean pharmacokinetic parameters and corresponding coefficient of variation (CV) following IV administration of 10 mg/kg EK-ECP to male rats are illustrated in Table 1. Those following oral administrations of 10, 100 and 1000 mg/kg EK-ECP to male rats are illustrated in Table 2.

#### 2.2.1. Group 1 (IV Bolus Administration)

Rats that received 10 mg/kg EK-ECP (Group 1) by IV bolus administration were exposed to 8,8′-bieckol, dieckol, and PFF-A. 8,8′-bieckol and dieckol plasma concentrations were quantifiable up to 36 h, whereas PFF-A plasma concentrations were only quantifiable up to 2 h post dose. Therefore, pharmacokinetic parameters derived for PFF-A in Group 1 should be interpreted with caution. Mean estimated concentrations at time 0 h (C_0_) were 7220 ng/mL, 8890 ng/mL, and 12.8 ng/mL for 8,8′-bieckol, dieckol, and PFF-A, respectively. Mean systemic exposure (AUC_Tlast_) values were 6620 ng·h/mL, 5290 ng·h/mL and, 7.81 ng·h/mL for 8,8′-bieckol, Dieckol, and PFF-A, respectively. Mean terminal half-life (T_1/2_) values were approximately 9.42, 11.9, and 0.731 h for 8,8′-bieckol, dieckol, and PFF-A, respectively, whereas effective half-life (Effective T_1/2_) values were 1.3, 1.48, and 0.36 h for 8,8′-bieckol, dieckol, and PFF-A, respectively. Mean clearance (Cl) values of 8,8′-bieckol and dieckol were similar at 162 and 193 mL/h/kg, respectively, and that of PFF-A was much greater at 84,000 mL/h/kg. The mean apparent volume of distribution (Vz) values of 8,8′-bieckol, and dieckol were 2200 and 3350 mL/kg, respectively, whereas that of PFF-A was much greater at 89,200 mL/kg. The mean apparent volume of distribution values estimated at steady state (Vss) were 366, 570, and 77,600 mL/kg for 8,8′-bieckol, dieckol, and PFF-A, respectively.

#### 2.2.2. Groups 2, 3, and 4 (Oral Administration)

Rats that received 10 mg/kg EK-ECP (Group 2) by oral gavage administration were not exposed to 8,8′-bieckol, dieckol, or PFF-A. All plasma concentrations were below the lower limit of quantitation in this group. Rats that received 100 mg/kg EK-ECP (Group 3) by oral gavage administration were only exposed to 8,8′-bieckol and dieckol. The plasma concentrations of 8,8′-bieckol and dieckol were quantifiable up to 12 and 8 h, respectively. All PFF-A plasma concentrations were below the lower limit of quantitation in this group. Rats that received 1000 mg/kg EK-ECP (Group 4) by oral gavage administration were exposed to 8,8′-bieckol, dieckol, and PFF-A. The plasma concentrations of 8,8′-bieckol and dieckol were quantifiable up to 36 h whereas those of PFF-A were only quantifiable up to 1 h post dose in a single rat.

Mean systemic exposure (AUC_Tlast_) and C_max_ values of 8,8′-bieckol were not determined at the 10 mg/kg dose and increased with increasing doses from 100 to 1000 mg/kg; a 1:10-fold increase in dose resulted in an approximate 1:71-fold increase in C_max_ and an approximate 1:84-fold increase in AUC_Tlast_ values. Mean systemic exposure (AUC_Tlast_) and C_max_ values of dieckol were not determined at the 10 mg/kg dose and increased with increasing doses from 100 to 1000 mg/kg; a 1:10-fold increase in dose resulted in an approximate 1:41-fold increase in C_max_ and an approximate 1:49-fold increase in AUC_Tlast_ values. Dose–exposure relationships could not be determined for PFF-A due to insufficient data (no quantifiable plasma concentration data at the 10 and 100 mg/kg dose groups). A single rat had measurable PFF-A plasma concentrations at the 0.25 and 0.5 h collection interval for the 1000 mg/kg dose group that resulted in a C_max_ value of 1.58 ng/mL.

Mean terminal half-life values and mean effective half-life values were not determined due to insufficient data for the 10 mg/kg dose group for 8,8′-bieckol and dieckol and for all dose groups for PFF-A. Mean terminal 8,8′-bieckol half-life values were 2.95 and 6.63 h for 100 and 1000 mg/kg dose groups, respectively, and mean terminal dieckol half-life values were 2.76 and 6.61 h for 100 and 1000 mg/kg dose groups, respectively. Mean effective 8,8′-bieckol half-life values were 2.24 and 4.41 h for 100 and 1000 mg/kg dose groups, respectively, and mean effective dieckol half-life values were 2.36 and 5.31 h for 100 and 1000 mg/kg dose groups, respectively.

#### 2.2.3. Bioavailability (Oral Versus IV Bolus Administration)

An assessment of exposure could not be made for rats following oral gavage administration of 10 mg/kg EK-ECP (all plasma concentrations were below the lower limit of quantitation). Bioavailability was determined for dieckol and 8,8-bieckol after a 100 and 1000 mg/kg oral dose of EK-ECP with a correction for differences in doses between the oral and IV administration routes. Bioavailability following oral gavage administration was approximately 0.069% to 0.5% for 8,8′-bieckol and 0.06% to 0.23% for dieckol.

## 3. Discussion

This study is the first to provide a comprehensive evaluation of the absorption, distribution, metabolism, and excretion (ADME) characteristics of eckols, a group of marine polyphenols with significant therapeutic potential. By examining the pharmacokinetic properties and bioavailability of key eckols—dieckol, 8,8′-bieckol, and PFF-A—following both intravenous and oral administration of EK-ECP to rats, we have gained valuable insights into how these compounds behave in vivo.

First, we confirmed the chemical composition of EK-ECP using the Folin–Ciocalteu assay method, which showed that the extract was 91.0% polyphenol. The ^1^H-NMR spectrum of EK-ECP, shown in Figure 2, displays peaks centered at 5.8 ppm (range: 5.5–6.9 ppm) and 9.2 ppm (range: 7.9–9.7 ppm), corresponding to the aromatic and phenolic hydroxyl protons of the phloroglucinol units found in dieckol, 8,8′-bieckol, and PFF-A. These NMR features highlight structural distinctions between phlorotannins (a.k.a., marine polyphenols) and common terrestrial polyphenols, such as flavonoids, flavanones, flavonols, and flavanols, which exhibit more complex proton environments due to their diverse chemical frameworks [69,70]. The hydroxyl protons observed in the phlorotannin structures are attached to aromatic rings, resulting in distinct downfield shifts (near 9 ppm) due to the electron-withdrawing effects of the aromatic system. This contrasts with the hydroxyl groups found in aliphatic alcohols in general. RP-HPLC analysis identified ten individual eckols within EK-ECP, including 8,8′-bieckol, dieckol and PFF-A—the three eckols chosen for pharmacokinetic monitoring. These three eckols were clearly separated in the HPLC chromatogram and represent different ranges of polarity within the phlorotannin extract, with 8,8′-bieckol being the most hydrophilic, dieckol in between, and PFF-A the most lipophilic.

In the pharmacokinetic study, rats receiving a 10 mg/kg dose of the extract via IV administration (Group 1) were exposed to 8,8′-bieckol, dieckol, and PFF-A. In contrast, rats that received the same dose orally (Group 2) showed no detectable levels of these eckols in their plasma. At a higher oral dose of 100 mg/kg (Group 3), only 8,8′-bieckol and dieckol were detectable, while PFF-A remained below the quantifiable limit. At the highest oral dose of 1000 mg/kg (Group 4), all three eckols were present in the plasma, although PFF-A was only measurable up to 1 h post dose in one rat.

The systemic exposure (AUC_Tlast_) and maximum concentration (C_max_) values for 8,8′-bieckol and dieckol could be determined at the 100 and 1000 mg/kg dose groups in a more than dose-proportional manner. However, due to limited data, dose–exposure relationships for PFF-A could not be established.

The bioavailability of 8,8′-bieckol was found to range from approximately 0.069% to 0.5%, and for dieckol, from 0.06% to 0.23%. This relatively low oral bioavailability poses a significant challenge for the development of these eckols as therapeutic agents administered orally. It underscores the need to explore advanced formulation strategies, such as nanoparticle delivery systems, lipid-based formulations, or the use of bioenhancers, as well as alternative routes of administration to improve systemic bioavailability.

Further research is also required to better understand the metabolic pathways involved in the first-pass effect and to identify potential metabolites that may contribute to the pharmacological activity of these eckols.

To address the potential reduction in bioavailability due to degradation by gastric juice, we evaluated the stability of dieckol as a representative compound of phlorotannins at a pH of 2 using UV–Vis spectroscopy. The results indicated no significant changes over a period of at least 5 h (unpublished data). Based on this, we interpret that gastric juice likely did not contribute meaningfully to the degradation of phlorotannins during the PK study.

Interestingly, despite their low bioavailability, the therapeutic efficacy of eckols, particularly PFF-A, which is virtually insoluble in water, has been demonstrated in numerous in vivo studies [13,25,26,37,45,47,50,51,53,54,60,61,64,65]. This suggests alternative distribution routes that could explain their in vivo effects, potentially involving interactions with the gut microbiome [71] or targeting lipid-rich tissues such as the lymphatic system and the central nervous system.

There is growing evidence that polyphenols can beneficially modulate the gut microbiota that play a key role in the pathogenesis of various chronic diseases, including cardiovascular disorders, metabolic syndromes, and neurodegenerative diseases [72]. Vazquez-Rodriguez et al. demonstrated that phlorotannins and polysaccharides from *Silvetia compressa* play a significant role in modulating the gut microbial ecosystem [73]. The hydroethanolic extract, rich in phlorotannins, promoted the growth of beneficial bacteria such as Bifidobacterium and Lactobacillus, while enhancing the synthesis of short-chain fatty acids (SCFAs), including acetic and propionic acids. Notably, key phlorotannins like eckstolonol and dieckol were metabolized within eight hours of fermentation; after 48 h, the extract achieved comparable effects to inulin in terms of bacterial growth and SCFA production [73]. These findings indicate the potential of phlorotannin-rich extracts in promoting gut health by favorably altering microbiota composition and metabolic activity. Given the limited systemic absorption of eckols following oral administration, it is plausible that their primary site of action lies within the gastrointestinal tract. Here, these compounds may interact with, and modify, the composition and activity of gut microbial communities. This microbial modulation could enhance gut barrier integrity, promoting a favorable immunological balance [74]. Through these mechanisms, eckols may exert therapeutic effects by supporting immune homeostasis and mitigating inflammatory responses. This proposed gut-focused pathway aligns with the findings from many in vivo studies, suggesting that the therapeutic potential of eckols may be mediated primarily through their interactions with the gut microbiome. Therefore, future studies should further investigate the impact of eckols on gut microbiota composition and function more systematically, as well as their downstream effects on host metabolism and immune responses.

Additionally, given the highly lipophilic nature of eckols, particularly PFF-A, they may preferentially accumulate in lipid-rich tissues offering another potential route of exerting therapeutic effects. Lipophilic compounds often bypass conventional absorption pathways, such as direct entry into the bloodstream, and instead enter the lymphatic system, which is rich in lipids [75,76]. This alternative pathway may facilitate the distribution of eckols to tissues like the central nervous system (CNS), which is protected by the lipid-rich blood–brain barrier. The CNS-directed effect of dieckol and PFF-A upon oral administration as low as 1.0 and 0.2 mg/kg/bw, respectively, in a mouse-model study [64], despite undetectable levels in plasma after oral administration in this study, suggests that eckols may utilize these lipid-based routes to exert their therapeutic effects. This could explain the observed efficacy in various in vivo models, despite their low systemic bioavailability. Targeting lipid-rich tissues, such as the lymphatic system and the CNS, could provide a therapeutic advantage in conditions where these tissues are involved such as neurodegenerative disorders [5] or systemic inflammation [77]. Exploring these distribution pathways in greater detail could reveal important insights into the pharmacodynamics of eckols and inform the development of novel therapeutic strategies that leverage their lipophilicity for targeted delivery.

The data from this study provide a crucial foundation for the future development of eckols as therapeutic agents. The ability of these compounds to achieve measurable systemic exposure after IV administration, combined with their established safety profile, supports their potential use in clinical settings. The pharmacokinetic parameters identified here can guide dosing strategies and help predict human pharmacokinetics, which are essential for advancing eckols-rich *E. cava* extracts toward clinical trials.

In this study, the pharmacokinetics of individual phlorotannins, such as dieckol, 8,8′-bieckol, and PFF-A, were assessed as components of a complex mixture within *E. cava* phlorotannins. It is important to note that the pharmacokinetic behavior of these compounds may differ when administered as part of this complex mixture compared to their administration as isolated molecules. When phlorotannins are part of a complex, their absorption, distribution, metabolism, and excretion (ADME) can be influenced by interactions with other phlorotannins in the mixture. These interactions may affect their solubility, competition for metabolic pathways, and excretion routes, potentially leading to altered bioavailability and systemic exposure. In contrast, administering these compounds as single molecules would provide a clearer understanding of their intrinsic pharmacokinetic properties, free from the influence of other components. This distinction highlights the complexity of interpreting pharmacokinetic data from complex natural extracts and underscores the need for further studies to explore the pharmacokinetics of individual phlorotannins in isolation.

In conclusion, this study significantly advances our understanding of the pharmacokinetic behavior of eckols. While challenges remain, particularly in enhancing oral bioavailability and in understanding their potential interactions with the gut microbiome, the findings provide a strong basis for further preclinical and clinical investigations aimed at harnessing the therapeutic potential of eckols, both through direct pharmacological effects and via modulation of the gut microbiome, for treating cancer and chronic degenerative diseases.

## 4. Materials and Methods

### 4.1. Materials

The raw material, *E. cava*, was freshly collected off the coast of Jeju Island, Korea. A voucher specimen has been deposited in the Laboratory of Aging and Degenerative Diseases at Hanbat National University, Daejeon, Korea, for future reference and authentication. Immediately after collection, the whole seaweed was thoroughly washed with tap water and air-dried. The dried algae were then cut into small pieces and stored in a dark room until use. *E. cava* phlorotannins (EK-ECP, also known as Seapolynol™) were prepared by Botamedi Inc. (Jeju, Korea) by extracting the pretreated raw material using 95% ethanol, according to a proprietary method.

Standard samples of individual phlorotannins for PK monitoring (dieckol (99.2%), 8,8′-bieckol (97.1%), and PFF-A (99.0%)) were provided by Botamedi Inc. (Jeju, Korea). Other standard samples for individual phlorotannins in EK-ECP that were not monitored during PK study, such as 2-O-(2,4,6-trihydroxyphenyl)-6,6′-bieckol, 6,6′-bieckol, 7-phloroeckol, 2-phloroeckol, eckol, dioxinodehydroeckol and fucofuroeckol A, were also provided by Botamedi Inc. (Jeju, Korea) at 95+% purity, and were used for the identification of notable minor peaks in the HPLC chromatogram of EK-ECP. Ultrapure water (18.2 MΩ·cm resistivity) was obtained from an in-house Millipore purification system (Milli-Q^®^, Merck Millipore, Burlington, MA, USA) and used for all experiments. Ethanol, propylene glycol, potassium phosphate monobasic, sodium phosphate dibasic, sodium chloride, HCl, methanol, acetonitrile, DMSO were obtained from ThermoFisher Scientific (Waltham, MA, USA). Formic acid, L-ascorbic acid, and indometacin were obtained from Sigma-Aldrich Co. (St. Louis, MO, USA).

### 4.2. Analytical Methods for Chemical Characterization of EK-ECP

^1^H NMR (500 MHz) was acquired on Bruker Avance III 500 MHz NMR spectrometer (Bruker Corporation, Billerica, MA, USA) using DMSO-*d*_6_ as a solvent. Chemical shifts were referenced to the residual solvent peaks (δ_H_ 2.50 for DMSO-*d*_6_). To quantify the total polyphenolic content of EK-ECP, the Folin–Ciocalteu reagent was reacted with EK-ECP dissolved in 50% aqueous ethanol, and the resulting solution was analyzed using a UV–Vis spectrophotometer (SPECTROstarNano, BMG Labtech, Ortenberg, Germany) at 725 nm. Quantification was performed by comparison to a calibration curve constructed with an anhydrous phloroglucinol standard over a concentration range of 0.04–0.20 µg/mL, demonstrating excellent linearity (R^2^ > 0.99). The method exhibited strong precision, with intra-day and inter-day relative standard deviations (RSD) below 2% across six replicates. Recovery studies confirmed the accuracy of the method, with recovery rates between 98% and 102% from spiked samples. Specificity was ensured, as no significant interference from other matrix components was observed at 725 nm. The reverse-phase HPLC chromatographic separation was achieved by running a gradient elution using Solvents A and B, optimized to resolve individual phlorotannins within the EK-ECP extract. The detailed HPLC conditions were as follows. The UV detector was set to a wavelength of 254 nm. A CAPCELL PAK C_18_ column (UG120, 5 μm) with dimensions of 4.6 mm × 250 mm was utilized. The mobile phase was delivered at a flow rate of 0.8 mL/min. The column temperature was maintained at 45 °C during the analysis. A sample volume of 10 μL of the EK-ECP solution was injected into the HPLC system for each run. The mobile phase composition was 0.05% acetic acid in water (Solvent A) and 100% Methanol (Solvent B). Individual phlorotannin peaks were identified by spiking EK-ECP with the corresponding standards and verifying the increase in the specific peak in the HPLC chromatogram.

### 4.3. Animals and Diets

Total of 20 male CD^®^ [Crl:CD^®^(SD)] rats (approximately 11.5 weeks of age) obtained from Charles River Laboratories (Portage, MI, USA) were used in our study. These included five rats with both jugular and femoral vein cannulations, and 15 rats with jugular vein cannulations. Of the 20 animals received, 16 male rats were placed in the study, comprising four rats with both jugular and femoral vein cannulations, and 12 rats with jugular vein cannulations. The rats were housed in the Mattawan facility of MPI Research Inc (currently Charles River Laboratories, Mattawan, MI, USA). Animal welfare for this study was in compliance with the U.S. Department of Agriculture’s (USDA) Animal Welfare Act (9 CFR Parts 1, 2 and 3). This facility maintained an animal welfare assurance statement with the National Institutes of Health Office of Laboratory Animal Welfare. All procedures were approved before the study by the institutional animal care utilization committee. Animals were individually housed and fed a basal diet of Lab Diet Certified Rodent Diet #5002, PMI Nutrition International, Inc. Food and water was available ad libitum except during fasting periods, when food was withheld, and during any required restraint periods, when both food and water were withheld. Animal rooms were maintained at (20 ± 2) °C and (50 ± 20)% relative humidity with a 12 h light/dark cycle throughout the study.

### 4.4. Dose Formulation and Dosing

For intravenous administration, EK-ECP was dissolved in 70/25/5 (*v*/*v*) phosphate-buffered saline (PBS)/propylene glycol/ethanol (pH 5.1 final concentration of 2 mg/mL) and stabilized at room temperature for 24 h. For oral administration, after an amount of EK-ECP appropriate to the final targeted formulation concentrations was weighed and placed in a bottle, a small amount of the vehicle (water for injection) was added and mixed using a vortex mixer and heated to between 60 to 70 °C until homogeneously suspended. Additional amounts of the vehicle were gradually added to yield the targeted EK-ECP concentrations (1, 10, and 100 mg/mL). Dosing occurred within 4 h after preparation completion. The individual constituent doses of dieckol, 8,8′-bieckol, and PFF-A in each dosing formulation were analyzed by RP-HPLC followed by interpolation of the corresponding area% to the calibration curve prepared with known concentrations of isolated standards. The dosing schedule is shown in Table 3. The animals were fasted for a minimum of 4 h prior to dosing and through the first 4 h of blood sample collection. Each animal in Group 1 received a single intravenous (IV) dose of the 10 mg/kg EK-ECP. The intravenous doses were administered as a slow bolus injection over 5 min through the femoral vein cannula. The cannula was flushed with approximately 0.3 mL of sterile 0.9% sodium chloride for injection, USP, immediately following dosing. Each animal in Groups 2, 3 and 4 received a single oral (PO) gavage dose of the appropriate test article formulation, as outlined Table 3. Oral gavage dosing formulations were continuously stirred throughout dosing.

### 4.5. Blood Sampling and Bioanlysis

Blood samples were collected pre dose and at 15 and 30 min and 1, 2, 4, 6, 8, 12 and 24 and 36 h post dose for each group. Blood samples were collected from the jugular vein cannula into syringes containing a stabilizer (ascorbic acid) and placed into tubes containing K_2_EDTA. The samples were centrifuged, and the resulting plasma was separated and immediately stored frozen at −50 to −90 °C until analysis. Acetonitrile precipitation and HPLC/MS/MS were used to determine the concentration of dieckol, 8,8′-bieckol and PFF-A from rat K_2_EDTA plasma with 2% ascorbic acid. An aliquot of the extract was injected onto an HPLC/MS/MS triple quadrupole mass spectrometer (Sciex API5500, MA, USA). A BetaBasic^TM^ HPLC column (2.1 × 50 mm) from ThermoFisher (Waltham, MA, USA) was used to separate dieckol, 8,8′-bieckol and PFF-A and the internal standard indomethacin from interfering compounds that may be present in the sample extract. The aqueous mobile phase was water with 0.1% formic acid solution (*v*/*v*), and the organic mobile phase was acetonitrile. The rinse solvent was 1:1 methanol: water. The peak area of the product ion of the compound (dieckol, 8,8′-bieckol and PFF-A) was measured against the peak area of the product ion of the internal standard. A calibration curve ranging from 1.00 ng/mL to 1000 ng/mL (eight concentrations in duplicate) was used to quantify dieckol, 8,8′-bieckol and PFF-A in the samples.

### 4.6. Pharmcokinetic Analysis

The mean and coefficient of variation (CV) were calculated for 8,8′-bieckol, dieckol, and PFF-A plasma concentrations at each time point. Concentrations less than the lower limit of quantitation (LLOQ, <1 ng/mL) were reported as, and set to, zero in the calculations. An individual plasma concentration–time profile was constructed for each animal per dose group from which pharmacokinetic parameters were derived.

The individual 8,8′-bieckol, dieckol, and PFF-A plasma concentration–time profiles from EK-ECP-dosed animals were analyzed using model-independent methods [78]. The analysis was performed with the aid of WinNonlin^®^ version 5.3, Microsoft^®^ Excel (versions 2003 and 2010) and SimgaPlot^®^ 12.

The following pharmacokinetic parameters were determined for each dose group: estimated concentration at time zero (C_0_) (Group 1 only); maximum observed plasma concentration (C_max_) (Groups 2, 3 and 4 only); time of maximum observed plasma concentration (T_max_) (Groups 2, 3 and 4 only); and area under the plasma concentration–time curve (AUC). The AUC from time 0 to 24 h (AUC_0–24h_), the AUC from time 0 to the time of the final quantifiable sample (AUC_Tlast_), and the AUC from time 0 to infinity (AUC_INF_) were calculated by the linear trapezoidal method. Half-life values (T_1/2_) were reported for each animal when the plasma concentration–time profile had sufficient plasma concentrations in the terminal elimination phase (at least 3 samples, not including T_max_) and an R^2^ of ≥0.9. Due to the observed multiphasic elimination, MRT_last_ and effective half-life values were also determined for each animal (effective T_1/2_ = (ln(2)⋅MRT_last_). Clearance (CL) (Group 1 only), apparent oral clearance (CL/F) (Groups 2, 3 and 4 only), volume of distribution (Vz) (Group 1 only), apparent volume of distribution after an oral dose (Vz/F) (Groups 2, 3 and 4 only), and volume of distribution at steady state (Vss) (Group 1 only) were calculated using the actual doses from each constituent of EK-ECP.

Measurable PFF-A concentrations at all PO dose levels were too few to allow for the accurate determination of pharmacokinetic parameters. Bioavailability (F) for 8,8′-bieckol, dieckol, and PFF-A could not be assessed for Group 2 (10 mg/kg PO) due to Group 2 plasma concentrations being below the lower limit of quantitation (LLOQ, <1 ng/mL). Bioavailability was determined for Groups 2 (100 mg/kg PO) and 3 (1000 mg/kg PO) for dieckol and 8,8′-bieckol, with appropriate dose adjustments. CL/F was determined directly as dose/AUC and Vz/F was determined directly as dose/(AUC_INF_).

## Figures and Tables

**Table 1:** Mean (CV) pharmacokinetic parameters following IV administration of 10 mg/kg EK-ECP to male rats.

Aanlyte ^a^	Actual Constituent Dose (mg/kg)	C_0_ (ng/mL)	T_last_ (h) ^b^	AUC_Tlast_ (ng·h/mL)	AUC_(0–24h)_ (ng·h/mL)	AUC_INF_ (ng·h/mL)	T_1/2_ (h)	MRT_last_ (h)	Effective T_1/2_ (h) ^d^	Cl (mL/h/kr)	Vz (mL/kg)	Vss (mL/kg)
8,8′-Bieckol	1.075	7220 (15)	36 (36–36)	6620 (6)	6540 (6)	6670 (6)	9.42 (9)	1.88 (5)	1.30 (5)	162 (6)	2200 (14)	366 (14)
Dieckol	0.91	8890 (18)	36 (36–36)	5290 (49)	5190 (49)	5390 (49)	11.9 (7)	2.14 (16)	1.48 (16)	193 (35)	3350 (36)	570 (28)
PFF-A	0.805	12.8 (31)	2 (1–2)	7.81 (38)	9.03 (36)	10.1 (30) ^c^	0.731 (4) ^c^	0.520 (27)	0.360 (27)	84,000 (26) ^c^	89,200 (29) ^c^	77,600 (30) ^c^

^a^ N = 4/analyte, unless otherwise noted, ^b^ Median (minimum-maximum), median only reported if collection interval, ^c^ N = 3; ^d^ Effective t1⁄2 calculated using mean residence time from time of dosing to time of last measurable concentration MRT_last_, using the calculation: ln(2)⋅MRT_last_.

**Table 2:** Mean (CV) pharmacokinetic parameters following PO administration of 10, 100 and 1000 mg/kg EK-ECP to male rats.

Aanlyte ^a^	Actual Constituent Dose (mg/kg)	C_max_ (ng/mL) ^b^	Tmax (h) ^d^	Tlast (h) ^d^	AUC_Tlast_ (ng·h/mL)	AUC_(0–24h)_ (ng·h/mL)	AUC_INF_ (ng·h/mL)	T1/2 (h)	MRT_last_ (h)	Effective T_1/2_ (h) ^j^	Cl/F (mL/h/kr)	Vz/F (mL/kg)
8,8′-Bieckol	0.92	0 (NC)	NA ^c^	NA	NR ^e^	NR ^e^	NR	NA	NA	NA	NA	NA
	10.3	11.2 (68)	(0.25–4)	8 (8–12)	37.7 (26)	41.5 (26)	44.0 (22) ^f^	2.95 (24) ^f^	3.23 (33)	2.24 (33)	242,000 (22) ^f^	1,060,000 (45) ^f^
	102.8	795 (37)	0.5 (0.5–0.5)	36 (36–36)	3170 (35)	3050 (35)	3210 (35)	6.63 (9)	5.93 (16)	4.11 (16)	35,100 (34)	342,000 (43)
Dieckol	0.27	0 (NC)	NA	NA	NR	NR	NR	NA	NA	NA	NA	NA
	8.79	6.67 (63)	(0.5–4)	8 (6–8)	24.3 (26)	27.2 (25)	31.4 (NC) ^g,h^	2.76 (NC) ^b,g^	3.40 (26)	2.36 (26)	287,000 (NC) ^b,g^	1,160,000 (NC) ^b,g^
	91.61	274 (42)	0.5 (0.5–0.5)	36 (36–36)	1200 (31)	1130 (30)	1230 (31)	6.61 (8)	7.67 (8)	5.31 (8)	80,000 (28)	768,000 (33)
PFF-A	0.41	0 (NC)	NA	NA	NR	NR	NR	NA	NA	NA	NA	NA
	4.17	0 (NC)	NA	NA	NR	NR	NR	NA	NA	NA	NA	NA
	41.33 ^i^	1.58	0.5	1	NR	NR	NR	NA	NA	NA	NA	NA

^a^ N = 4/analyte, unless otherwise note, ^b^ NC= Not calculated, CV not calculated when mean plasma concentration is 0 ng/mL, ^c^ NA = Not applicable; ^d^ Median (minimum-maximum), median only reported if collection interval; ^e^ NR = Not reported, (Area under the plasma concentration time curve (AUC) could not be reported due to less than 3 consecutive quantifiable concentrations)**;** ^f^ N = 3, ^g^ N = 2, ^h^ NC = Not calculated, CV is not calculated when N < 3, ^i^ N = 1; ^j^ Effective t_1⁄2_ calculated using mean residence time from time of dosing to time of last measurable concentration [MRT_last_], using the calculation: ln(2)⋅MRT_last._

**Table 3:** Dosing schedule.

Group	Treatment	Dose Route	EK-ECP Content in Dose Formulation (mg/mL)	Dose Volume (mL/kg)	EK-ECP Dose Level (mg/kg)	Constituent Dose Level (mg/kg) ^a^	Number of Rats
1	EK-ECP	IV	2	5	10	1.075/0.91/0.805	4
2	EK-ECP	PO	1	10	10	0.92/0.27/0.41	4
3	EK-ECP	PO	10	10	100	10.3/8.79/4.17	4
4	EK-ECP	PO	100	10	1000	102.8/91.61/41.33	4

^a^ Constituent dose levels presented for 8,8′-bieckol/dieckol/PFF-A.

## Data Availability

The original data presented in the study are included in the article; further inquiries can be directed to the corresponding author.
